# Natural language processing for suicidal behavior detection in short Spanish clinical notes from first-contact interviews

**DOI:** 10.3389/fninf.2026.1919962

**Published:** 2026-10-07

**Authors:** Juan Carlos Villagomez-Garcia, Juan Martínez-Miranda, Ansel Y. Rodríguez-González

**Affiliations:** Unidad Acadèmica Tepic, Centro de Investigación Científica y de Educación Superior de Ensenada, Tepic, Nayarit, Mexico

**Keywords:** data augmentation, first-contact clinical text, lexical analysis, machine learning, Spanish-language transcripts, suicidal behavior detection

## Abstract

**Introduction:**

Suicide is a multifactorial phenomenon influenced by social, cultural, biological, and psychological factors, whose early detection remains challenging. According to the World Health Organization, suicide is the third leading cause of death among individuals aged 15–29 years, representing a major public health concern for young populations worldwide. In first-contact clinical settings, screening opportunities are often constrained by limited time, sparse patient information, and operational workflows. This study evaluates the feasibility of detecting suicidal behavior from short Spanish-language texts collected during initial screening interviews.

**Methods:**

The dataset comprises 374 anonymized records labeled as “no suicidal behavior,” “ideation,” “planning,” or “attempt,” with a strong imbalance toward “attempt,” reflecting operational clinical usage rather than population-representative sampling. To address data scarcity, we applied data augmentation methods (Easy Data Augmentation for Spanish, backtranslation, and large language model-based synthesis), validated through statistical analysis and clinician-based plausibility assessments.

**Results:**

Exploratory analyses revealed highly constrained and overlapping lexical patterns across classes, indicating the challenging nature of the task. A Support Vector Machine achieved 0.53 macro *F*_1_ in binary classification (“suicidal behavior” vs. “no behavior”), while BETO, a Spanish BERT model, reached 0.36 macro *F*_1_ in multiclass classification. Under the same text-only conditions, human specialists achieved macro *F*_1_ scores of 0.29 and 0.15 respectively; however, these results were based on only 20 real texts evaluated with double annotation across five specialists and should therefore be interpreted cautiously.

**Discussion:**

Although performance is modest compared to studies using full clinical records, these findings suggest that short-text machine learning could support nonspecialists in first-contact settings. Future work should expand datasets and explore multimodal approaches, including voice and text, to improve clinical applicability and early detection.

## 1 Introduction

Mental health is a complex and multifaceted domain, with suicide representing one of its most critical challenges due to the interaction of numerous social, cultural, biological, psychological, and environmental factors (Goel et al., 2023; Khan and Mian, 2010; Gunnell and Lewis, 2005; Masango et al., 2008; Bertolote et al., 2003). Contextual factors—including family environment, socioeconomic status, access to healthcare, and the presence of chronic or psychiatric conditions—further shape an individual's risk profile, resilience, and coping capacity (Argothy Almeida et al., 2024).

Early identification of suicidal behavior is essential, as delayed detection contributes substantially to preventable morbidity and mortality (Horowitz et al., 2009). This issue is particularly relevant among young people, given that suicide is the third leading cause of death among individuals aged 15–29 years (WHO, 2025). Evidence indicates that many individuals consult healthcare providers within the month preceding a suicide attempt (Schwartz-Lifshitz et al., 2012; De Leo et al., 2013; Luoma et al., 2002; Pirkis and Burgess, 1998), yet only a fraction are correctly recognized as at-risk during routine visits (Claassen and Larkin, 2005). First-contact and emergency healthcare settings therefore represent pivotal opportunities for early detection, but these settings are often constrained by limited consultation time, insufficient mental health training among general medical staff, and restricted access to comprehensive patient records (Downey et al., 2012; Kerrison and Chapman, 2007; Al-Nofaiey et al., 2022; Gordon, 2012).

Recent advances in natural language processing (NLP) and machine learning provide promising avenues for detecting suicidal behavior from unstructured clinical text (Velupillai Sumithra et al., 2019; Fernandes et al., 2018). In addition, digital tools may encourage disclosure, as patients sometimes feel more comfortable sharing sensitive information with automated systems than with human clinicians (Branley-Bell et al., 2023). In contrast to most NLP-based approaches for suicidal behavior detection, which rely on longitudinal electronic health records (EHRs), clinical notes, or multi-modal patient data, this work focuses exclusively on minimal, first-contact textual inputs. The proposed approach assumes no prior clinical history, no structured variables, and no follow-up information, reflecting realistic screening scenarios such as initial interviews, online intake forms, or early telehealth interactions. By restricting the input to short, free-text responses collected at first contact, this study addresses a complementary and underexplored setting in suicidal behavior assessment, where early signals must be detected under severe informational constraints.

In this study, we present a controlled feasibility and benchmarking investigation of text-only, first-contact suicidal behavior screening in Spanish clinical contexts. The contributions are four-fold:

1. We constructed and analyzed a curated dataset of brief, first-contact medical notes in Spanish, capturing the informational constraints typical of frontline screening scenarios.
2. We systematically evaluated multiple text augmentation strategies under extreme class imbalance, assessing their statistical significance and impact on clinically relevant performance metrics.
3. We experimented with classical machine learning and transformer-based models, characterizing achievable performance levels in a low-resource, high-risk classification setting.
4. We performed a controlled, text-only comparison between automated models and human specialists, providing insight into the relative capabilities and limitations of each under identical information constraints.

The research questions that guided this study are the following:

- **RQ1**: what level of performance, measured by macro-*F*_1_ score and recall can be achieved by automated models for the identification of suicidal behaviors under strict first-contact conditions using exclusively textual information?
- **RQ2**: to what extent can different data augmentation and modeling strategies reduce the performance gap between automated systems and clinical specialists in this constrained setting? To address these questions, the remainder of the paper is organized as follows. Section 2 reviews related work on the automatic detection of suicidal behavior. Section 3 describes the characteristics and analysis of the collected data, while Section 4 presents the data augmentation and synthetic data generation procedures. Section 5 details the classification models evaluated in this study. Section 6 compares the performance of the automated approaches with that of medical specialists. Finally, Section 7 discusses the main findings, and Section 8 presents the conclusions and directions for future research.

## 2 Related work

The identification of suicidal behavior has been extensively explored in recent years through a variety of approaches, including both machine learning and deep learning techniques, and utilizing combinations of categorical, numerical, and textual data. For instance, Nordin et al. (2021) employed K-nearest neighbors (KNN), support vector machines (SVM), and Naive Bayes algorithms with data from patients diagnosed with depression. Their study used medical history records to determine which patients were at risk of suicide, achieving the highest accuracy (92%) with tree ensemble methods. Notably, this work relied exclusively on categorical data extracted from clinical histories and did not incorporate textual information.

Textual data has also been leveraged in the study by Zhu et al. (2020), who applied TF, IDF, and TF-IDF representations to electronic health records, combined with decision trees, random forests, SVM, and AdaBoost. This approach achieved an *F*_1_ score of up to 98.9% in a binary classification of patients exhibiting suicidal behaviors. Several other studies have processed textual data by applying toolkits that extract and encode specific concepts from clinical narratives, subsequently transforming them into categorical variables for model training. Models such as random forests, classification and regression trees (CART), and other decision tree variants have been employed, with varying performance. For example, Gkotsis et al. (2016) reported an accuracy of up to 65%, while Su et al. (2020) and Carson et al. (2019) reported recall rates around 60%.

Other strategies have aimed at enhancing predictive performance through tailored model designs. Barak-Corren et al. (2017) stratified the data by gender and developed a Bayesian classifier for each subgroup, achieving 95% accuracy in binary suicidal behavior classification. Cusick et al. (2021) employed Word2Vec embeddings and a convolutional neural network (CNN) to classify clinical notes, reaching 98.4% accuracy. More recently, (Singh Rawat and Yu 2022) fine-tuned a transformer-based pretrained model, ScANER, for classification tasks, achieving 91% accuracy on a self-constructed dataset derived from ScAN. Across these approaches, performance gains are strongly associated with the availability of rich contextual information, such as longitudinal electronic health records, structured clinical variables, or detailed narrative notes collected over multiple encounters. In many cases, models operate on aggregated patient histories that implicitly encode prior diagnoses, symptom progression, and clinician interpretation. As a result, reported metrics primarily reflect performance under conditions where substantial contextual and temporal information is available.

Despite the methodological diversity and the different types of data used across these studies, a common feature is the availability of extensive datasets. In almost all cases, researchers had access to complete medical records, providing rich information per patient, including prior diagnoses, symptoms, treatments, physicians' notes, and discharge summaries. To date, no study has focused on first-contact data, i.e., initial screening interviews with short textual notes that may or may not lead to a mental health disorder diagnosis. This distinction highlights the unique contribution of the present study, which addresses a gap in the literature by targeting minimal, early-stage textual data for suicidal behavior assessment.

## 3 Exploratory data analysis

### 3.1 Data description

The dataset employed in this study originates from a computational platform presented in Martínez-Miranda et al. (2019, 2020), an integrated system for screening and longitudinal monitoring of patients exhibiting suicidal behaviors. The platform consists of a web interface for clinicians and a mobile application incorporating a conversational agent to facilitate patient interaction as a complement to therapeutic treatment. The dataset was not constructed through a sampling strategy designed to approximate population-level variability or class balance. Instead, it constitutes an *operational census* of all patients processed by the deployed screening platform over a fixed multi-year observation period of approximately five years, beginning at the time the system was integrated into routine clinical practice (April 29, 2019–August 20, 2024). As such, the corpus reflects real-world clinical usage rather than a statistically curated or population-representative cohort.

The screening platform is embedded within a clinical workflow and is primarily intended as a frontline triage and decision-support tool. Consequently, the observed class distribution reflects the case mix induced by referral policies, institutional intake procedures, and frontline usage patterns, rather than an intentionally balanced design. This operational context explains both the high prevalence of suicidal behavior labels and the limited number of negative cases in the dataset. The collected dataset comprised 374 anonymized records. Participants had a mean age of 29.63 years and standard deviation (SD) = 12.35, with 236 cases (63%) corresponding to individuals aged 30 years or younger, indicating a predominance of adolescents and young adults in the sample. The dataset included 278 (74%) female and 96 (26%) male participants. Each record was assigned to one of four diagnostic categories reflecting the severity of suicidal behavior: *no suicidal behavior, suicidal ideation, suicide planning*, or *suicide attempt*. The class distribution was highly imbalanced, comprising 11 records labeled as *no suicidal behavior*, 36 as *suicidal ideation*, 48 as *suicide planning*, and 279 as *suicide attempt* (Figure 1 illustrates this distribution). Diagnostic labels were assigned by mental health specialists following the first-contact clinical interview and the administration of validated assessment instruments, including the Plutchik Suicide Risk Scale (Plutchick et al., 1989). Consequently, the labels reflect expert clinical judgment supported by standardized suicidal behaviors assessment procedures.

**Figure 1:**
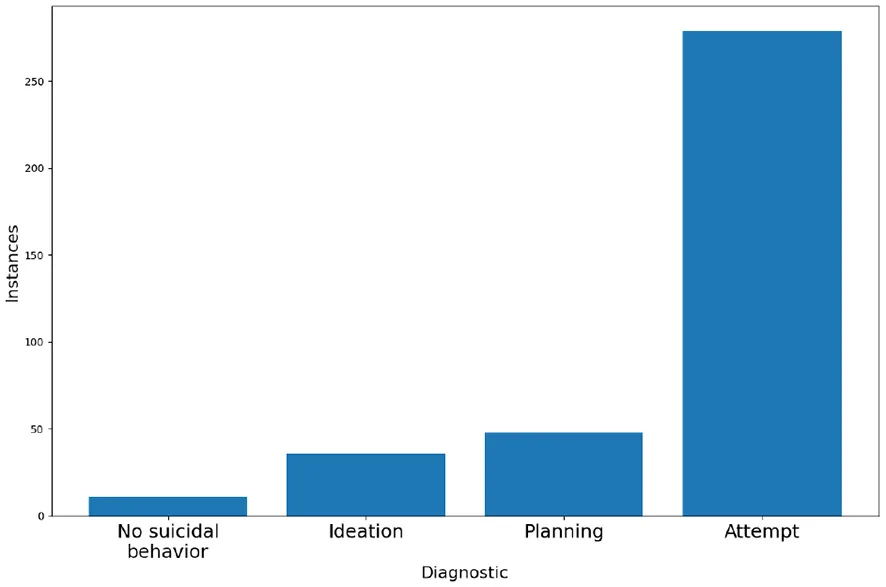
Distribution of classes on the dataset.

All records reflect highly constrained, sparse, and heterogeneous textual descriptions typical of frontline screening scenarios. This extreme class imbalance is an inherent property of the deployment setting rather than a design choice. The platform's clinical integration introduces an inherent selection bias by preferentially capturing cases following suicide-related clinical intervention. This mechanism inflates the observed prevalence of suicidal behavior and limits direct generalization to broader patient populations or lower-risk screening contexts, such as primary care or community-based settings. From a statistical perspective, the critically small number of negative samples constrains the stability of class-specific performance estimates and the reliability of confidence intervals, particularly for precision, recall, and macro-averaged metrics associated with the non-suicidal class. Small absolute changes in prediction outcomes can therefore result in large relative fluctuations in these measures. Accordingly, performance results in this study are interpreted as conditional on the operational distribution induced by the clinical workflow, and emphasis is placed on comparative model behavior and relative performance trends rather than on absolute metric values.

The findings of this study are framed as a controlled feasibility and benchmarking analysis under strict text-only, first-contact conditions in a clinical environment. The results are not intended to support claims of prevalence estimation, population-level screening performance, or immediate deployment readiness in broader or lower-risk populations. Generalization beyond institutional contexts with similar referral pathways, screening protocols, and documentation practices should therefore be made with caution. All records analyzed in this study were fully de-identified and originated from the dataset described by Martínez-Miranda et al. (2020). Because the present study involved the secondary analysis of anonymized data, informed consent from participants was not required. No information that could directly or indirectly identify individuals was available to the researchers.

After the initial extraction, all texts were processed using an automated spell checker to correct typographical errors. The cleaning procedure was applied uniformly across the entire corpus; no records were removed or modified beyond orthographic corrections (an example can be seen in Table 1). To characterize the dataset and determine whether note length represented a systematic difference among classes that could potentially influence subsequent text-based classification, we calculated the mean word count and corresponding standard deviation for each class (Table 2). Because word counts were non-normally distributed and exhibited unequal variances across classes, a Kruskal–Wallis H test was used to compare note-length distributions, revealing an overall significant difference among the four groups (*p* = 0.0013). A *post hoc* Dunn's test with Bonferroni adjustment (Figure 2) showed that only the comparison between the “ideation” and “attempt” groups remained significant (*p* < 0.005), whereas all other pairwise comparisons were non-significant.

**Table 1:** Examples of the collected short texts.

Screening question	Patient's response (Spanish, Original)	Patient's response (English, translated)
Patient's Objective (Of the treatment).	Ponerse bien	To feel better
Patient's self-concept.	Enojona, explosiva y berrinchuda	Angry, explosive and prone to tantrums
Patient's Main fears.	Perder a mi hermano y a mi mam	Losing my brother and mother
Patient's premorbid personality.	Muy inteligente, enojona y berrinchuda	Intelligent, angry and prone to tantrums
Patient's opinion on same sex.	Unas son buenas y otras malas	Some are good, others are bad
Patient's opinion on opposite sex.	Unos buenos y otros malos	Some are good, others are bad

**Table 2:** Mean and standard deviation (SD) of each class' length in words.

Class	Mean (μ)	SD (σ)
No behavior	32.82	15.96
Ideation	41.97	16.17
Planning	38.67	18.12
Attempt	32.29	13.54

**Figure 2:**
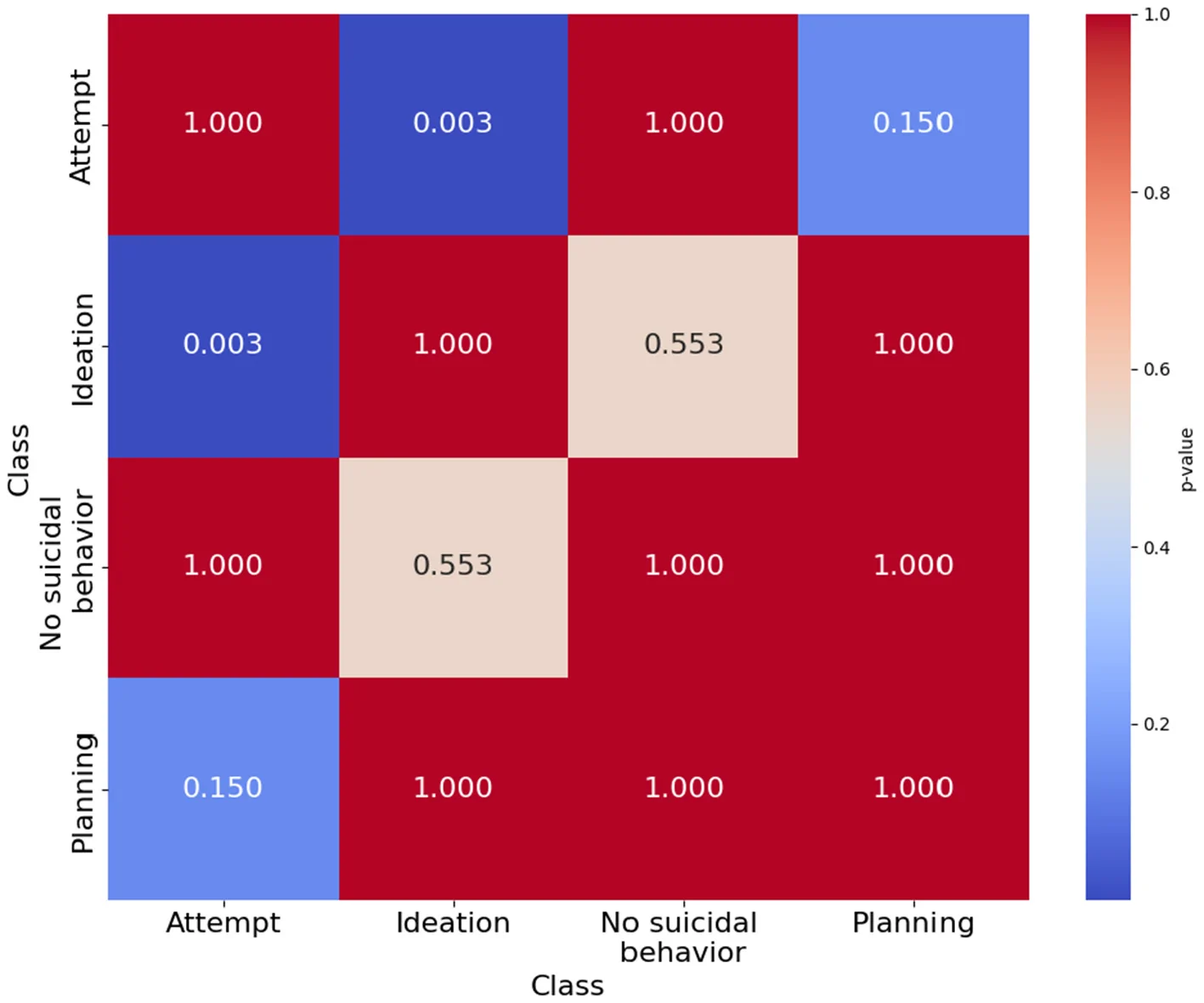
Dunn heatmap of suicidal behavior categories where dark red (high *p*-values) represent no significant differences.

Thus, note length did not consistently differentiate the suicidal behavior classes and instead provides additional evidence of the substantial overlap among them. This analysis was included as an exploratory characterization of the corpus and to assess whether text length could constitute an obvious class-related feature or potential confounding factor. Note length was not explicitly included as a control variable, covariate, or independent feature in the subsequent modeling analyses. Accordingly, the classification experiments relied on the textual representations rather than on explicit adjustment for note length. These findings also provide limited support in our dataset for previously reported behavioral distinctions based on text characteristics, such as those described by RamÃrez-Cifuentes et al. (2020), and therefore note-length differences were not considered a defining characteristic of the diagnostic groups in subsequent analyses.

### 3.2 Wordclouds

Word clouds were generated to illustrate the most frequent terms under a binary classification: *no behavior* vs. *any behavior*. For the negative class, common terms included *paciente* (patient), *hombre* (man), and *ninguno* (none). For the positive class, frequent terms included *persona* (person), *alegre* (happy), *solo* (alone/lonely), and *bien* (good/fine), as shown in Figures 3a b.

**Figure 3:**
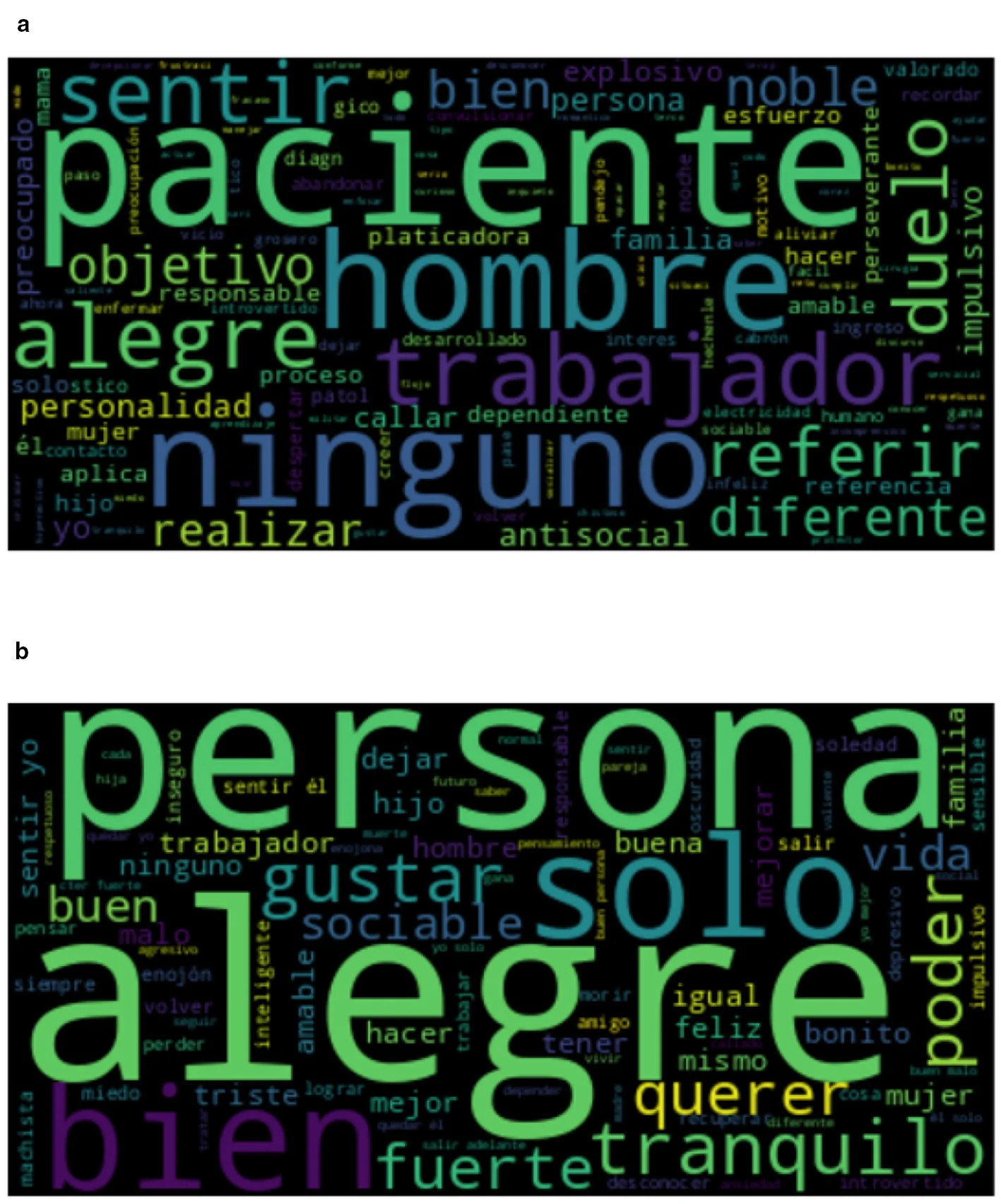
WordClouds of classes. **(a)** Wordcloud for negative class. **(b)** Wordcloud for positive class.

### 3.3 Jaccard index

As a complementary analysis, the Jaccard index was computed to quantify the degree of vocabulary overlap between classes. This metric measures the proportion of shared terms between two sets, where lower values indicate greater dissimilarity and, consequently, a higher potential for lexical separability. For the binary separation, the Jaccard index yielded a value of 0.0511, suggesting that the classes share approximately 5.1% of their total vocabulary. The total vocabulary comprised 1,789 unique words, of which 87 were shared between classes. The minority class (*no suicidal behavior*) contained only 118 words in total, leaving 31 unique terms exclusive to this class. The overlap and number of unique words per binary class are illustrated in the Venn diagram shown in Figure 4a. A similar pattern was observed among the suicidal behavior classes: *ideation* contained 120 unique words, *planning* included 169, and *attempt* accounted for 950 out of the total 1,671 words. The overlap among these classes is depicted in Figure 4b.

**Figure 4:**
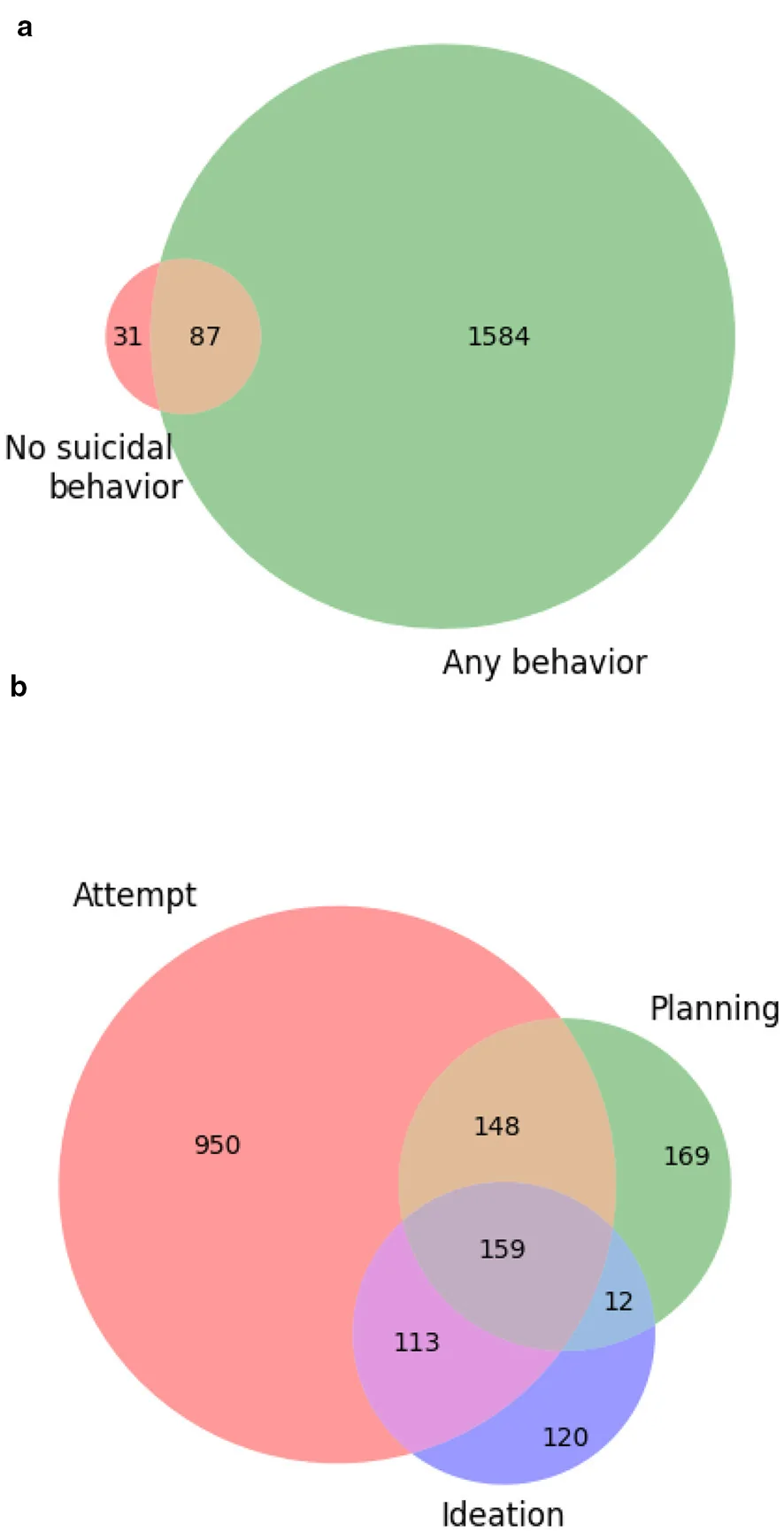
Venn figures for the different classes. **(a)** Binary. **(b)** Multiclass. **(a)** Venn diagram of overlap between suicidal and non-suicidal behavior classes. **(b)** Venn diagram of overlap between suicidal behavior classes.

### 3.4 t-SNE visualization

To obtain a low-dimensional view of the lexical space, t-distributed stochastic neighbor embedding (t-SNE) (Maaten and Hinton, 2008) was applied to high-dimensional vectors based on Spanish GloVe embeddings. Sentence-level vectors were constructed by averaging token embeddings and projected into two- and three-dimensional spaces (Figures 5a, 6a). Because mean pooling neglects syntactic structure and word order, we also conducted a secondary visualization experiment using the BETO transformer model, which preserves these features (Figures 5b, 6b). In all visualizations, the data points are highly intermingled, and no discernible cluster boundaries can be identified. This lack of separation indicates that the semantic signatures of the four classes are not visually distinct within the examined embedding spaces. Such results suggest that the classification task is non-trivial and potentially challenging for machine learning algorithms. Moreover, since t-SNE emphasizes local neighborhood structure, the presence of locally mixed labels further indicates that the embeddings capture similar linguistic patterns across diagnostic categories.

**Figure 5:**
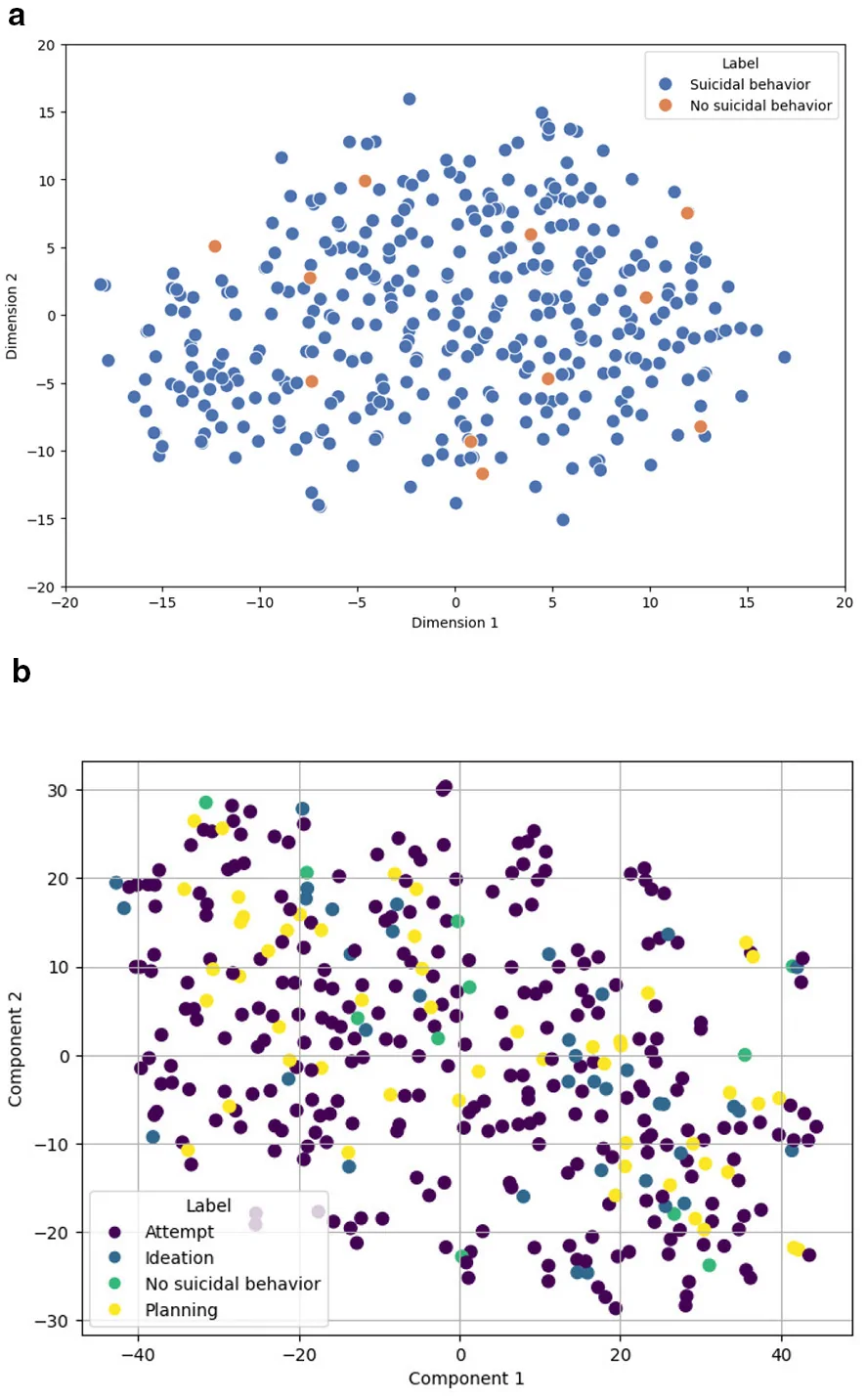
2D t-SNE representation. **(a)** Binary. **(b)** All classes. **(a)** 2D t-SNE representation of binary classes. **(b)** 2D t-SNE representation of all classes.

**Figure 6:**
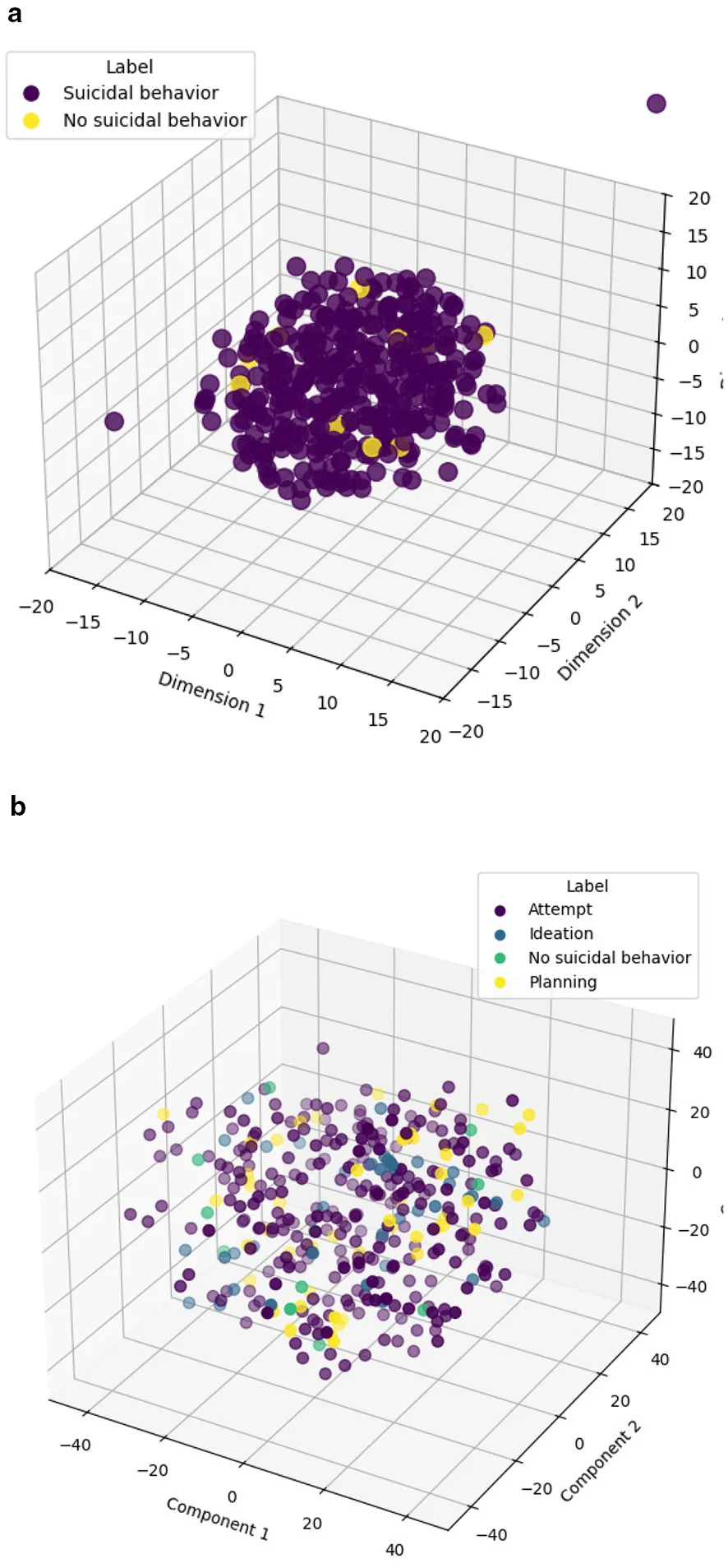
3D t-SNE representation. **(a)** Binary. **(b)** All classes. **(a)** 3D t-SNE representation of binary classes. **(b)** 3D t-SNE representation of all classes.

## 4 Data augmentation and synthesis

The original corpus contained a limited number of samples with relatively short responses, reflecting the constraints of first-contact clinical documentation. This data scarcity limits the stability of modern machine learning and transformer-based models, which typically benefit from training regimes involving datasets in the order of thousands of instances. Consequently, several data augmentation and synthesis strategies were employed to increase the dataset's size and linguistic diversity, while preserving the diagnostic structure of the original corpus.

### 4.1 Augmentation and generation strategies

#### 4.1.1 LLM-based easy data augmentation (EDA)

The Easy Data Augmentation (EDA) framework proposed by Wei and Zou (2019) was adapted for this study. EDA applies four elementary text transformations—synonym replacement, random insertion, random swap, and random deletion—to generate new instances from existing samples. The original implementation relies on WordNet, an English lexical database, which is unsuitable for Spanish clinical text. To address this limitation, the method was reimplemented using large language models (LLMs). Specifically, Llama 3 70B and GPT-4o were prompted to perform the EDA operations directly in Spanish, preserving grammatical structure and clinical tone. This procedure resulted in an augmented corpus approximately three times larger than the original dataset.

#### 4.1.2 Back-translation

A back-translation procedure was applied in which each text was translated into an intermediate language and subsequently translated back into Spanish. Using Google Translate and nine randomly selected intermediate languages, this approach generated paraphrases that preserve semantic content while introducing syntactic and lexical variability.

#### 4.1.3 One-shot synthetic generation

One-shot synthetic examples were generated using GPT-4o following a controlled prompting protocol. The model was instructed to generate short Spanish-language patient responses simulating first-contact clinical interviews, conditioned on a target diagnostic category (no suicidal behavior, ideation, planning, or attempt). Prompts provided a single exemplar per class and required the model to generate responses to a fixed set of intake-style questions (e.g., treatment motivation, self-perception, and primary fears), preserving semantic and contextual coherence while avoiding repetition of the exemplar content. Generated texts were constrained to be brief, written in the first person, and clinically neutral, and the model was explicitly instructed not to add explanations or external context. This procedure yielded approximately 7,000 synthetic instances. During post-processing, a subset of generated samples was removed because they contained non-Spanish output, malformed text, or structurally inconsistent responses. The resulting distribution of real and synthetic examples per class is illustrated in Figure 7a.

**Figure 7:**
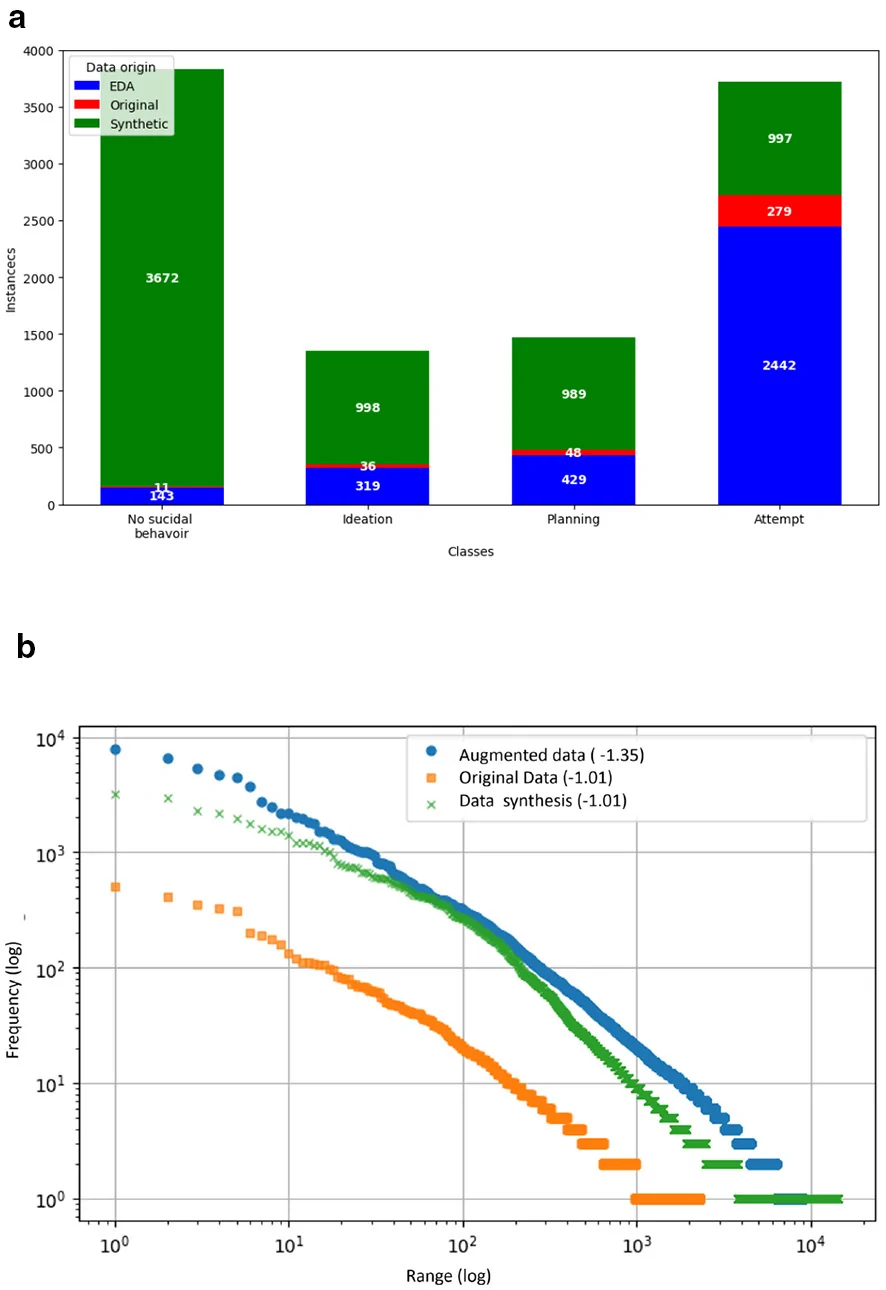
Characteristics of new data distribution. **(a)** New distribution of classes after data augmentation and synthesis; and **(b)** Zipf Graph, log-log scale.

### 4.2 Validation of created samples

Given the clinical nature of the domain, validation of augmented and synthetic texts was designed to address two complementary aspects: *linguistic realism* and *diagnostic fidelity*. These analyses provide descriptive evidence that synthetic data are suitable for model training, without making unsupported claims about absolute clinical validity.

#### 4.2.1 Lexical distribution analysis

First, we examined whether the augmented and synthesized corpora preserve characteristic lexical statistics of naturally occurring texts. Following Zipf's law (Zipf, 2013, 2016), the frequency *f* of a word is expected to be inversely proportional to its rank *r* ($f\propto \frac{1}{r}$), resulting in a log-log plot that approximates a straight line with slope close to −1. The corpus was partitioned into three subsets: original data, augmented data (EDA and back-translation), and fully synthetic data. For each subset, word frequencies were computed and ranked, and the results plotted on a log-log scale (Figure 7b). All three distributions exhibit approximately linear behavior, indicating that both augmentation and generation procedures preserve global Zipfian word-frequency patterns. This analysis is descriptive and captures surface-level lexical properties; it does not guarantee semantic coherence or diagnostic correctness of individual texts.

#### 4.2.2 Blind plausibility assessment

To assess whether generated texts resemble realistic patient responses, a blind evaluation was conducted with five licensed clinical psychologists. A balanced set of 40 texts (20 original and 20 synthetic) was randomly sampled. Each psychologist evaluated 16 texts (eight real and eight synthetic), and each text received two independent ratings. Reviewers classified each text as *plausible* or *implausible* in terms of whether it could realistically reflect a patient's response in a first-contact mental health interview. This assessment focused on surface-level realism rather than diagnostic accuracy.

#### 4.2.3 Label-consistency protocol

Plausibility alone does not ensure that a synthetic instance preserves its intended diagnostic label. To address this, clinicians performing the plausibility assessment were additionally asked to assign one of the four diagnostic categories (*no suicidal behavior, ideation, planning*, or *attempt*) to each evaluated text based solely on its content, without access to the intended label. This procedure served as a qualitative check of label consistency. While no formal inter-rater agreement metrics were computed, reviewers' observations indicated that the majority of synthetic examples were aligned with their intended diagnostic categories. Therefore, this check provides descriptive evidence that the augmented and synthetic corpus maintains sufficient diagnostic fidelity to support downstream model training.

Together, the aforementioned validation procedures provide complementary evidence that synthetic texts are both linguistically realistic and broadly consistent with their intended labels. Although these analyses do not guarantee clinical validity for individual instances, they support the use of synthetic data as a methodological tool to mitigate scarcity in the training set while avoiding contamination of evaluation data.

### 4.3 Impact of synthetic data on model training

Because the number of synthetic samples substantially exceeds the number of original clinical records, there exists a risk that models may overfit to stylistic or structural regularities introduced by the generation process rather than to the underlying clinical distribution. To evaluate the contribution of each augmentation strategy, an ablation protocol was defined in which models are trained and evaluated under three conditions:

1. Original data only,
2. Original data with EDA and back-translation,
3. Original data with full synthetic augmentation.

Comparative performance across these settings is reported in Section 5, allowing the incremental effect of each augmentation component to be quantified and isolating potential performance gains attributable specifically to large-scale synthetic generation.

### 4.4 Ethical and methodological considerations

The use of large language models to generate mental health—related text introduces specific ethical and technical risks, including unintended memorization of sensitive patterns, hallucination of clinically implausible content, and reinforcement of cultural or diagnostic stereotypes. In this study, synthetic data were used exclusively for methodological evaluation and model training, and were strictly excluded from validation and test partitions containing real clinical records. The validation protocols described above were designed to ensure that synthetic text serves as a methodological tool for mitigating data scarcity rather than as a substitute for real patient data or clinical judgment.

## 5 Classification tasks

Using the processed dataset, the classification stage was structured into two sequential tasks. The first task, *identification*, corresponds to a binary decision problem aimed at determining the presence or absence of suicidal behavior in a given text. The second task, *classification*, is a multiclass problem in which all positive instances identified in the first stage are assigned to one of the three clinical categories: ideation, planning, or attempt. Both tasks were evaluated using a five-fold cross-validation protocol applied at the record level. Performance was computed independently on each fold and subsequently aggregated to obtain average metrics. This procedure was adopted to mitigate the effect of sample partitioning on the reported results and to provide a measure of stability under repeated train—test splits, rather than to support formal statistical inference at the population level.

For both the binary and multiclass settings, complete classification reports were generated, including confusion matrices, precision, specificity, and overall accuracy. However, the primary quantitative indicators reported in this study are per-class recall (sensitivity) and the macro-averaged *F*_1_ score. Recall is emphasized due to the high clinical cost associated with false negatives, while macro *F*_1_ assigns equal weight to each class and therefore counterbalances the strong class imbalance present in the dataset, particularly the scarcity of negative examples. Finally, we explicitly note that the limited number of real negative samples constrains the statistical stability of performance estimates for that class. As a result, the reported metrics should be interpreted as indicative of relative model behavior under the defined experimental protocol rather than as definitive estimates of generalization performance in broader clinical populations.

### 5.1 Identification

For the identification task (binary discrimination between suicidal and non-suicidal behavior), we adopted a Support Vector Machine (SVM). This choice was based on a systematic preliminary comparison involving multiple classical machine learning classifiers together with the transformer-based BETO model (Cañete et al., 2020), all evaluated under the same cross-validation protocol and successive experimental settings. The evaluated architectures and principal hyperparameters are summarized in Table 3, while the corresponding Macro-*F*_1_ scores obtained during the model selection process are presented in Table 4. Although both classical machine learning models and BETO were evaluated under identical data partitions and augmentation settings, SVM consistently exhibited the most robust performance under the final binary experimental protocol and was therefore selected for the detailed analyses reported in this work. The reported SVM results correspond to the best-performing configuration identified during this comparative evaluation, which combined LLM-based EDA, back-translation, and one-shot synthetic generation applied exclusively to the training data.

**Table 3:** Architectures and hyperparameters evaluated during the preliminary model selection for binary suicidal behavior identification.

Family	Model	Text representation	Architecture/hyperparameters
Classical ML	Gaussian Naïve Bayes	Average of 300-dimensional Spanish GloVe embeddings	Default Scikit-learn implementation; variance smoothing = 10^−9^.
Classical ML	Random Forest	Average of 300-dimensional Spanish GloVe embeddings	100 decision trees; Gini impurity; bootstrap enabled; class_weight=None.
Classical ML	Support Vector Machine	Average of 300-dimensional Spanish GloVe embeddings	RBF kernel; regularization parameter *C* = 1; remaining parameters kept at default values.
Deep Learning	LSTM	300-dimensional Spanish GloVe embeddings	Maximum sequence length = 50 tokens; embedding layer initialized with pretrained GloVe and frozen; LSTM layer with 16 units; tanh activation; recurrent dropout = 0.20; Dense output layer with Softmax; categorical cross-entropy loss; AdamW optimizer; learning rate 10^−5^; early stopping.
Transformer	BETO	BETO tokenizer	Pretrained BETO (Spanish BERT); Dense output layer with two neurons; Softmax activation; categorical cross-entropy loss; AdamW optimizer; learning rate 10^−5^; 5 training epochs.

**Table 4:** Preliminary evaluation of candidate classifiers for binary suicidal behavior identification (Macro-*F*_1_).

Model	Original	Original + augmentation	Original + synthetic	Synthetic only	Filtered synthetic	Final protocol
Random forest	0.48	0.49	0.49	0.45	0.52	0.52
Gaussian Nave Bayes	0.49	0.53	0.47	0.46	**0.53**	**0.53**
SVM	0.49	0.49	**0.67**	0.41	0.52	**0.53**
LSTM	–	0.50	0.46	0.46	0.46	0.51
BETO	–	**0.54**	0.57	0.30	0.45	0.52

Bold values indicate the higher Macro-F1 score obtained by each classifier for each dataset.

Each document was first converted into a fixed-length sentence vector by averaging pre-trained Spanish GloVe embeddings (300-dimensional token vectors). Given that the negative class (“non-suicidal”) is severely underrepresented in the original data, all real negative instances were reserved exclusively for evaluation. Synthetic negative samples were incorporated only into the training set to augment the minority class while avoiding contamination of the test distribution. The remaining samples were divided into five folds for cross-validation to obtain an unbiased estimate of model performance. Crucially, all augmented instances were included exclusively in the training partitions and were excluded from both validation and test folds. This separation ensured that no information leakage occurred that could artificially inflate the reported performance metrics.

The SVM was then trained with the parameters: C=1, Kernel = Linear, and the rest of the parameters left to default. For this task, the Support Vector Machine (SVM) achieved a macro *F*_1_-score of 0.53 and an overall accuracy of 0.86. The recall was 0.09 for the negative class and 0.97 for the positive class. The remaining performance metrics are presented in Table 5. Additionally, the all-fold average confusion matrix was computed to determine the behavior of the classifier, which can be observed in Figure 8a. The confusion matrix indicates that the SVM tends to favor the majority class. This was an expected behavior, as during the exploratory data analysis, it was discovered that the data appeared to have no discernible separation, independent of the label.

**Table 5:** Classification report for binary classification.

Metric	No suicidal behavior	Suicidal behavior
Precision	0.37	0.87
Recall	0.09	0.97
Accuracy	0.86	
*F*_1_ score (macro)	0.53	

**Figure 8:**
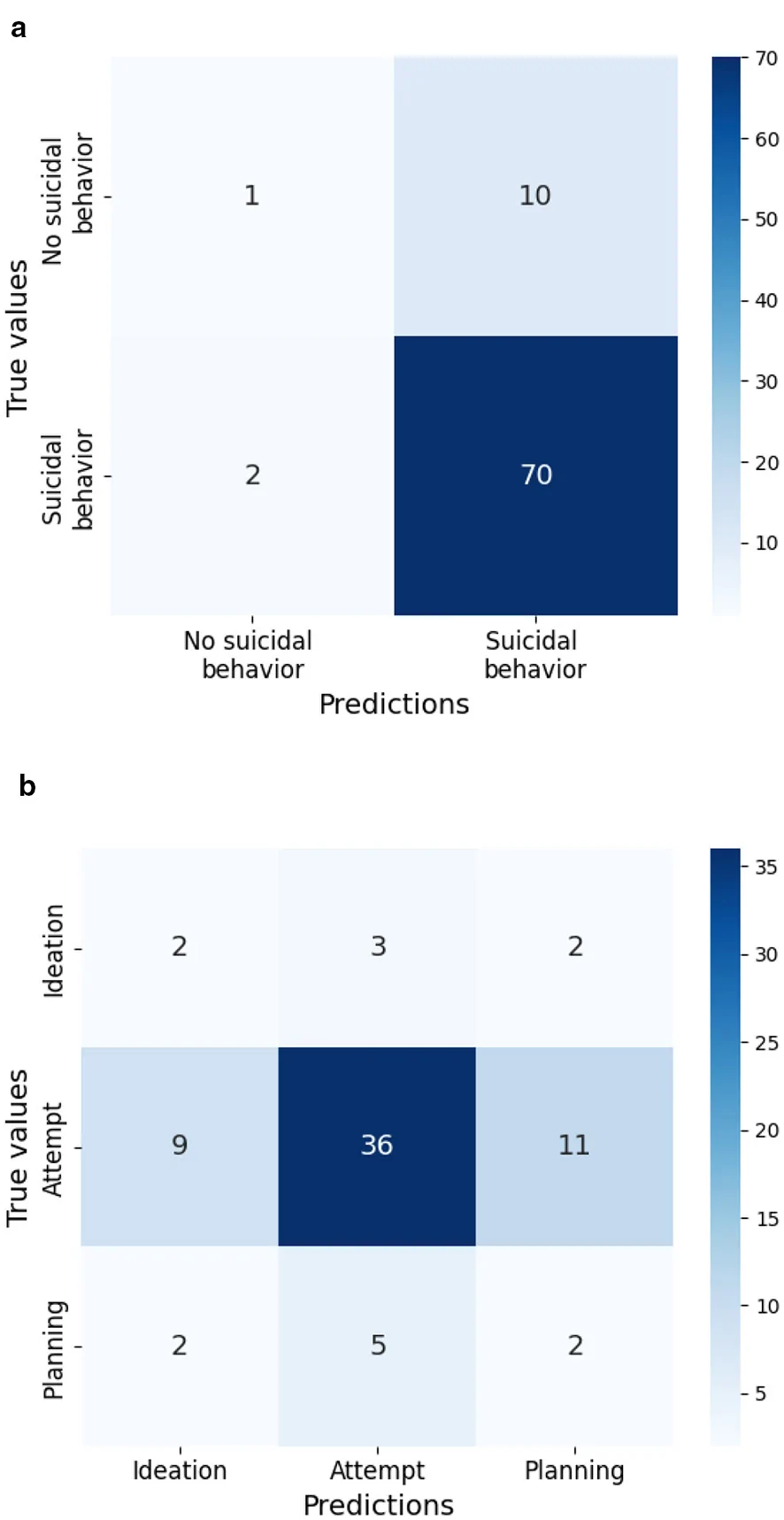
Best average confusion matrix. **(a)** Binary. **(b)** All classes. **(a)** Average confusion matrix of SVM. **(b)** Confusion matrix of multiclass classification.

### 5.2 Classification

For the multiclass classification task, the same cross-validation protocol as in the binary setting was employed. A five-fold split was applied, and all synthetic or augmented instances were included exclusively in the training folds. No such examples appeared in validation or test partitions, thereby preventing information leakage. Several classical machine learning classifiers together with the transformer-based BETO model were comparatively evaluated under identical data partitions and augmentation settings. The evaluated architectures and principal hyperparameters are summarized in Table 6, while the corresponding Macro-*F*_1_ scores obtained during the model selection process are presented in Table 7. Among all evaluated alternatives, BETO consistently achieved the strongest overall performance under the final multiclass experimental protocol and was therefore selected for the detailed analyses presented in this paper.

**Table 6:** Architectures and hyperparameters evaluated during the preliminary model selection for multiclass suicidal behavior classification.

Family	Model	Text representation	Architecture/hyperparameters
Classical ML	Gaussian Naïve Bayes	Average of 300-dimensional Spanish GloVe embeddings	Default Scikit-learn implementation; variance smoothing = 10^−9^.
Classical ML	Random Forest	Average of 300-dimensional Spanish GloVe embeddings	100 decision trees; Gini impurity; bootstrap enabled; class_weight=None.
Classical ML	Support Vector Machine	Average of 300-dimensional Spanish GloVe embeddings	RBF kernel; regularization parameter *C* = 1; remaining parameters kept at default values.
Deep Learning	LSTM	300-dimensional Spanish GloVe embeddings	Maximum sequence length = 50 tokens; pretrained frozen GloVe embedding layer; LSTM with 16 units; tanh activation; recurrent dropout = 0.20; Dense output layer with Softmax adapted to multiclass prediction; categorical cross-entropy loss; AdamW optimizer; learning rate 10^−5^; early stopping.
Transformer	BETO	BETO tokenizer	Pretrained BETO (Spanish BERT); Dense output layer with four neurons (multiclass output); Softmax activation; categorical cross-entropy loss; AdamW optimizer; learning rate 10^−5^; 5 training epochs.

**Table 7:** Preliminary evaluation of candidate classifiers for multiclass suicidal behavior classification (Macro-*F*_1_).

Model	Original	Original + Augmentation + Synthetic	Final protocol
Random Forest	0.21	0.28	0.28
Gaussian Nave Bayes	0.26	0.29	0.29
SVM	0.27	0.31	0.31
LSTM	0.31	0.34	0.34
BETO	**0.36**	**0.36**	**0.36**

Bold values indicate the higher Macro-F1 score obtained by each classifier for each dataset.

To adapt BETO to our task, we appended a single dense classification head to the pooled CLS token output. The head consisted of a linear layer with three units, corresponding to the number of positive behavior classes, followed by a softmax activation, yielding class probabilities. BETO and its classification head were jointly fine-tuned on each training fold using the standard cross-entropy loss. Training employed an AdamW optimizer (initial learning rate = 2*10^−5^), a linear warm-up schedule over the first 10% of steps, batch size= 8, a maximum of 10 epochs, and early stopping based on validation loss with a patience of 3.

BETO obtained an accuracy of 0.73 and a macro *F*_1_ score of 0.36, the rest of the metrics are shown in Table 8. As with binary classification, the all-fold average confusion matrix was computed and graphed, and is shown in Figure 8b. The confusion matrix indicates that BETO was unable to accurately distinguish among the different class labels, exhibiting misclassifications across all categories. Similar to the binary classification results, this outcome was anticipated by both the t-SNE representation and the Venn diagram illustrating vocabulary overlap.

**Table 8:** Classification report for multiclass classification.

Metric	Ideation	Planning	Attempt
Precision	0.08	0.26	0.80
Recall	0.03	0.24	0.90
Accuracy	0.73		
*F*_1_ score (macro)	0.36		

## 6 Comparison against mental health specialists

After an exhaustive review of related studies, we found that previous works—such as those by Murikipudi et al. (2023), Shorten et al. (2021), and Nordin et al. (2021)–rely on data modalities substantially different from those used in the present study. For instance, earlier research typically analyzes complete electronic health records (EHRs), incorporating categorical, textual, or multimodal data. In contrast, our corpus consists exclusively of first-screening free-text patient interview transcripts. These methodological differences make direct comparisons of performance metrics impractical. Therefore, an alternative evaluation strategy was implemented by comparing the results with those provided by specialists operating under the same text-only information constraints.

To establish a reference point for the second research question, five mental health specialists with prior experience using the suicidal behavior screening platform where the textual data were obtained Martínez-Miranda et al. (2020) were recruited. Following an approach similar to the earlier evaluation of synthetic text viability, a balanced set of 40 interview transcripts was prepared, comprising 20 original and 20 synthetically generated texts. These transcripts were randomly assigned such that each specialist reviewed 16 transcripts (eight original and eight synthetic), and each transcript received two independent assessments. Reviewers were instructed to assess suicidal behavior based solely on the information contained in each transcript and to classify each case into one of four categories: no suicidal behavior, suicidal ideation, suicidal planning, or suicide attempt. This assessment procedure differs from routine clinical practice, in which clinicians typically integrate visual cues, patient history, and standardized assessment instruments alongside patient narratives. The restriction to textual information was intentionally imposed to mirror the same input modality available to the automated models and to reflect the study's objective of evaluating text-based decision support under minimal-information conditions.

Answers were collected and processed using the same evaluation pipeline applied to the classification algorithms. For quantitative reporting, only responses corresponding to real (non-synthetic) texts were considered. The results were analyzed in two settings: (i) a binary approximation, in which all positive behaviors were grouped into a single suicidal class, and (ii) a full multiclass setting, in which all four diagnostic categories were retained. In the binary setting, the specialists achieved an overall accuracy of 0.30 and a macro-averaged *F*_1_ score of 0.29. The complete classification report is presented in Table 9, and the corresponding confusion matrix is shown in Figure 9a. When the responses were analyzed in the full multiclass setting, the specialists obtained an accuracy of 0.25 and a macro-averaged *F*_1_ score of 0.15. The remaining class-wise metrics are reported in Table 10, and the associated confusion matrix is shown in Figure 9b. Visual inspection of the confusion matrix suggests a tendency to underestimate the existence of suicidal behavior when only textual information is available, particularly for intermediate categories such as ideation and planning. This observation highlights the limitations of relying exclusively on free-text responses for fine-grained clinical differentiation.

**Table 9:** Specialists classification report for binary classification.

Metric	No suicidal behavior	Suicidal behavior
Precision	0.24	0.67
Recall	0.80	0.13
Accuracy	0.30	
*F*_1_ score (macro)	0.29	

**Figure 9:**
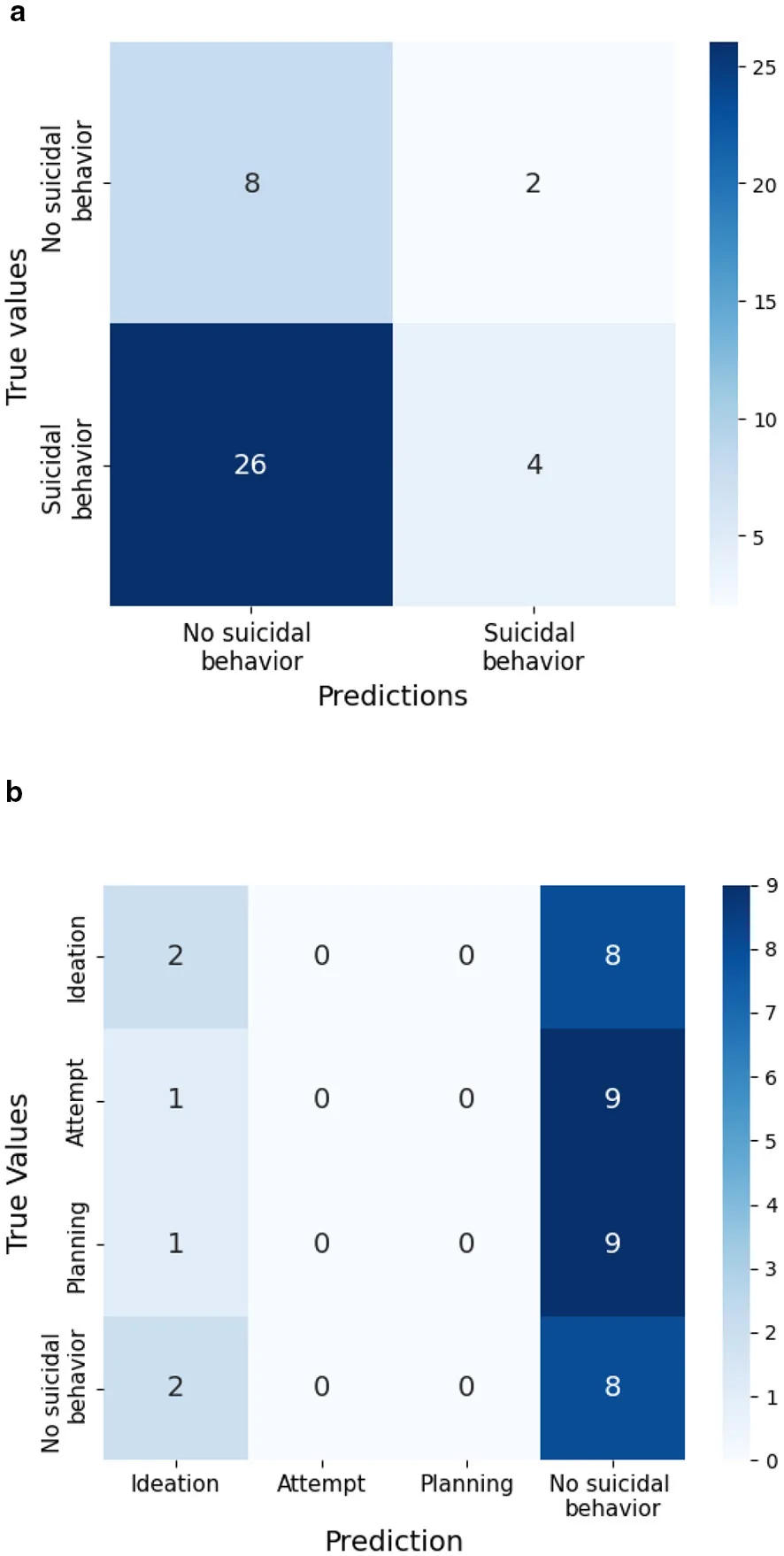
Specialists' confusion matrix. **(a)** Binary. **(b)** All classes. **(a)** Confusion matrix of specialists, binary setting. **(b)** Confusion matrix of specialists, multiclass setting.

**Table 10:** Specialists classification report for multiclass classification.

Metric	No behavior	Ideation	Planning	Attempt
Precision	0.33	0.00	0.00	0.24
Recall	0.20	0.00	0.00	0.80
Accuracy	0.25			
*F*_1_ score (macro)	0.15			

Under these highly constrained, text-only conditions, the automated models achieved higher macro-averaged *F*_1_ scores than the specialists on this limited evaluation set. Specifically, in the binary task, the models reached a macro *F*_1_ of 0.53 compared to 0.29 for the clinicians, and in the multiclass task, the BETO model achieved a macro *F*_1_ of 0.36 compared to 0.15 for the specialists. These results should not be interpreted as evidence that the proposed models surpass clinical expertise. The small number of evaluated samples, the absence of inter-rater reliability analysis, and the artificial nature of the task–restricted to isolated text without access to patient history, clinical observation, or standardized instruments such as the Beck Scale for Suicide Ideation (Beck et al., 1979)—substantially limit the clinical generalizability of this comparison. Instead, this experiment is intended to illustrate relative model and specialist behavior within a narrowly defined, minimal-information screening scenario, which may be representative of certain emergency or primary care contexts where comprehensive assessment tools and specialized training are not readily available.

## 7 Discussion

This study was inherently exploratory and was designed as a controlled feasibility and benchmarking analysis of text-only, first-contact suicide behavior screening in Spanish clinical settings. Its primary objective was not to propose a deployable diagnostic system, but to characterize the performance limits of automated models under the severe informational constraints faced in frontline screening scenarios. Many general practitioners may have limited formal training in suicidal behaviors assessment and may rely primarily on short, protocol-driven screening questions. Consequently, any potential deployment of the proposed approach must be undertaken with caution and a clear understanding of its limitations. The system is not intended to replace formal psychiatric evaluation or clinical judgment. Instead, it should be viewed as a decision-support tool designed to identify individuals who may be at elevated risk and to facilitate timely referral to mental health specialists for comprehensive assessment and intervention. Such a tool could be integrated into a variety of digital health platforms across different levels of care and clinical specialization, particularly in settings where patient interviews are routinely documented or transcribed. In these contexts, the system could provide automated alerts that prompt further mental health evaluation, thereby supporting early identification and improving access to specialized care.

In this context, the research questions formulated in the Introduction can be addressed as follows:

1. **(RQ1) What level of performance, measured by macro-F_1_** **score and recall can be achieved by automated models for the identification of suicidal behaviors under strict first-contact conditions using exclusively textual information?**

Under the experimental protocol and dataset constraints considered in this work, the highest observed performance was obtained by the SVM model in the binary identification task, achieving a macro-F_1_ score of 0.53, an overall accuracy of 0.86, and a recall of 0.97 for the binary suicidal-behavior class. In the multiclass classification task, the BETO model achieved a macro-F_1_ score of 0.36 and an accuracy of 0.73. These observed macro-F_1_ scores and class-specific recall values for suicidal behavior categories may be regarded as practical upper bounds on the performance of automated models in a first-contact screening context that relies exclusively on textual information. While these values remain limited in absolute terms, they reflect the severe information bottleneck imposed by the absence of longitudinal patient history, behavioral observation, and standardized clinical assessment instruments. The results suggest that, within this low-resource setting, further improvements are more likely to depend on the availability of richer or multimodal input data and larger, more diverse training corpora than on incremental changes in model architecture alone.

2. **(RQ2) To what extent can different data augmentation and modeling strategies reduce the performance gap between automated systems and clinical specialists in this constrained setting?**

The controlled comparison with mental health specialists operating under the same text-only constraints indicates that the combination of data augmentation and transformer-based modeling substantially narrows the performance gap. In the binary screening task, the best-performing automated model attains macro-F_1_ values that exceed those obtained by the specialists, while in the multiclass task the performance becomes comparable. These findings suggest that, when clinical context is intentionally removed, modeling and augmentation strategies can partially compensate for the resulting loss of information, supporting the role of automated systems as early-stage screening aids rather than replacements for expert clinical judgment.

From a clinical risk perspective, the binary SVM model achieved a high overall accuracy (0.86); however, this value should not be interpreted as evidence of balanced classification performance. The recall of only 0.09 for the No Suicidal Behavior class indicates that the model has a very limited ability to correctly recognize patients without suicidal behavior under the current decision threshold, largely as a consequence of the extreme class imbalance. In contrast, recall for the suicidal behavior class reached 0.97, reflecting a deliberate bias toward minimizing false negatives, which are clinically more critical than false positives. Consequently, the proposed model should not be used as a standalone diagnostic or exclusion tool. Instead, it is intended as a decision-support mechanism to assist clinicians in identifying patients who may require further assessment. In practical deployments, the operating decision threshold could be adjusted according to the intended clinical application to achieve different sensitivity-specificity trade-offs. For screening scenarios, a conservative threshold favoring high sensitivity may be preferable, whereas other applications may require a more balanced operating point. Under either configuration, automated predictions should remain embedded within a human-in-the-loop workflow in which flagged cases trigger referral to trained mental health professionals rather than autonomous clinical decisions.

Moreover, there is a concern in the construction of the binary training set. Because only eleven authentic non-suicidal cases were available, all of them were intentionally reserved for evaluation, and the classifier learned the negative class exclusively from GPT-4o-generated synthetic examples. This design avoided contamination of the evaluation set and ensured that all reported results were obtained on authentic clinical data. Nevertheless, it also implies that the learned decision boundary for the negative class depends on the representativeness of the synthetic examples with respect to real non-suicidal narratives. Consequently, the principal limitation is not necessarily an optimistic bias in the reported performance, but uncertainty regarding the external validity and generalizability of the learned boundary. Furthermore, by withholding all authentic negative examples from training, the experimental protocol intentionally imposed a more stringent learning scenario, so the reported binary performance should be interpreted as a conservative estimate under extreme data scarcity rather than as evidence of deployment-ready performance.

## 8 Conclusions and future work

This work evaluated the potential of text-only machine learning approaches to detect suicidal behavior from short, first-contact Spanish medical notes. Despite extreme class imbalance and limited textual information, the models showed promising performance in identifying suicidal behavior. In this evaluation, the SVM achieved a macro F1 of 0.53 for binary detection, compared with 0.29 for clinicians, while BETO achieved a macro F1 of 0.36 for multiclass classification, compared with 0.15 for clinicians. However, given the small number of real texts evaluated by specialists and the absence of formal statistical significance testing or confidence intervals, these differences should be interpreted cautiously and should not be considered conclusive evidence of superior model performance. Overall, the findings suggest that even brief textual records may contain meaningful cues that could support early screening in clinical contexts, although larger studies and formal statistical comparisons are needed to confirm the robustness of these results.

Nonetheless, the study highlights several inherent limitations. The operational dataset reflects a clinical workflow rather than a population-representative sample, which restricts generalizability. Furthermore, high lexical overlap and semantic sparsity across categories challenged multiclass classification, limiting the discrimination among ideation, planning, and attempt labels. Model performance was also sensitive to data scarcity and augmentation strategy, emphasizing the need for careful validation when incorporating synthetic samples.

An additional limitation is that the binary classifier learned the non-suicidal class exclusively from synthetic examples, as all authentic negative cases were reserved for evaluation. While this conservative protocol prevented evaluation contamination, it also limits confidence in the learned representation of the negative class. Future work should prioritize collecting substantially larger numbers of authentic non-suicidal first-contact records to determine whether the observed performance generalizes when both training and evaluation rely exclusively on real clinical data.

Several directions for future research emerge from the present study, taking advantage of available opportunities while recognizing the constraints associated with data acquisition. First, further evaluations are needed to establish comprehensive performance baselines for both mental health specialists and general practitioners, thereby enabling a more nuanced comparison between human assessments and automated models operating under text-only, first-contact conditions. In addition, future studies should investigate alternative model architectures and training strategies for both binary and multiclass classification tasks. This includes exploring additional data augmentation and synthesis approaches, such as the use of different large language models, latent-space transformations, and adversarial techniques, with the aim of improving model robustness and generalization.

As new patient records become available through routine platform use, models can be incrementally retrained and updated to accommodate evolving data distributions. Although dataset size and class balance depend primarily on platform adoption and patient flow rather than factors under direct researcher control, continuous model refinement may enhance predictive performance over time. Finally, future work should explore multimodal approaches that combine textual information with complementary sources of data, including sociodemographic characteristics and voice features. Such cross-modal frameworks may improve predictive accuracy and increase the clinical utility of automated screening systems in low-resource, first-contact settings.

## Data Availability

The data analyzed in this study is subject to the following licenses/restrictions: The dataset is part of a computational platform for screening of mental health problems licenced for its use to the health services of Nayarit (Mexico). Requests to access these datasets should be directed to jmiranda@cicese.edu.mx.
